# The Impact of Tumor Microenvironmental Acidity on Bicalutamide Sensitivity in Prostate Cancer Cells

**DOI:** 10.1155/proc/4321914

**Published:** 2026-04-05

**Authors:** Pedram Golmohammadi, Idris Haghani, Iman Menbari Oskouie, Rahil Mashhadi, Ahmadreza Rezaeian, Seyed Mohammad Kazem Aghamir

**Affiliations:** ^1^ Urology Research Center, Tehran University of Medical Sciences, Tehran, Iran, tums.ac.ir; ^2^ Department of Urology, Yale School of Medicine, New Haven, Connecticut, USA, yale.edu

**Keywords:** acidic, androgen deprivation, bicalutamide, neutral, pH, prostate cancer

## Abstract

**Background:**

Prostate cancer is the second most commonly diagnosed cancer worldwide. Although androgen deprivation therapy initially demonstrates clinical benefit, disease relapse with more aggressive phenotypes frequently occurs. The acidic tumor microenvironment in solid tumors may alter drug responsiveness. This study investigates how extracellular pH influences the cytotoxic effects of bicalutamide in human prostate cancer cell lines.

**Methods:**

PC3 and LNCaP cells were exposed to bicalutamide at varying concentrations at pH 7.4 and pH 6.8. IC50 values were determined using the MTT assay. Cell migration, apoptosis, and cell cycle distribution were evaluated by wound‐healing assay, annexin V/PI staining, and DNA content analysis, respectively. The expression of *BAX*, *BCL2*, *E-cadherin*, *N-cadherin*, *SNAI1*, *AR*, and *VEGF-C* was quantified by qPCR.

**Results:**

Bicalutamide (140 μg/mL) reduced PC3 cell viability to 39.62% at pH 7.4 compared with 51.36% at pH 6.8. In LNCaP cells, viability declined to 33.64% at pH 7.4% and 56.09% at pH 6.8. Treated PC3 cells exhibited significantly greater migration at pH 6.8 (*p* < 0.01). Early apoptosis in treated LNCaP cells was significantly reduced at pH 6.8 (*p* < 0.001). Both cell lines demonstrated enhanced S phase accumulation and reduced G1‐phase distribution at pH 6.8. The *BAX*/*BCL2* ratio was significantly decreased at pH 6.8, indicating the suppression of proapoptotic signaling. Additionally, genes associated with epithelial–mesenchymal transition (EMT) were upregulated, and *VEGF-C* and *AR* expression increased at pH 6.8 (*p* < 0.05).

**Conclusion:**

The efficacy of bicalutamide in prostate cancer cells is significantly influenced by extracellular pH. The drug exerts stronger cytotoxic, antimigratory, and proapoptotic effects at physiological pH (7.4) compared with acidic conditions (6.8).

## 1. Introduction

Prostate cancer is identified as the second most commonly diagnosed cancer and the fourth leading cause of cancer‐related mortality among men worldwide, with 1,466,680 new diagnoses and 396,792 deaths recorded in 2018 [1]. The androgen receptor (AR), a ligand‐activated transcription factor, is essential for the progression of prostate cancer [2, 3]. Although second‐generation AR antagonists, including enzalutamide, apalutamide, and darolutamide, have demonstrated improved survival outcomes in patients with prostate cancer, they do not offer a definitive cure [4]. Complete inhibition of the androgen pathway poses challenges, as reduced androgen levels can promote cancer cell proliferation, whereas elevated androgen levels are associated with cell cycle arrest and differentiation [5]. Treatment with AR antagonists (flutamide or bicalutamide) represented the standard systemic therapy [6]. Prolonged androgen deprivation therapy caused evidence of pathological regression [7], but relapse with a more aggressive form of prostate cancer has been observed [8]. This relapse has been termed hormone‐refractory, castration‐resistant, or androgen‐independent prostate cancer [9–11]. Overcoming this resistance is crucial for improving therapeutic outcomes in prostate cancer treatment [12]. Indeed, the development of more effective therapies has to be considered a priority for the worldwide biomedical community.

Human tumors differ from normal tissues, both morphologically and physiologically. Compared to normal tissues, tumors often show abnormal vascular architecture and function, including vessel blind ends, loss of hierarchy, and increased permeability [13]. These abnormalities cause irregular and inadequate perfusion, resulting in insufficient O_2_ supply [14], making hypoxia a common tumor feature [15]. Tumor cells adapt by switching to glycolysis even under normal oxygen levels, known as the Warburg effect, leading to increased glucose use and lactic acid production, and resulting in strong hypoxia, low glucose, high lactate, and extracellular acidosis with a pH below 6.5 [14]. This acidic environment drives metabolic reprogramming in cancer cells [16], including a reduction in intracellular pH. Cancer cells export protons to maintain an alkaline intracellular environment, supporting proliferation and survival [17]. Methods such as microelectrodes and pH‐sensitive fluorescent dyes indicate that tumor extracellular pH typically ranges from 6.5 to 6.9 [18], with highly metastatic areas measuring 6.1 to 6.4 and nonmetastatic tumors measuring 6.7 to 6.9 [19]. These findings suggest a relationship between acidic tumor microenvironments and aggressiveness, linking them to increased metastatic potential, especially in prostate cancer [20]. Few studies have examined how altering pH affects drug efficacy and cytotoxicity in cancers [21–23], though low extracellular pH in tumors can reduce the effectiveness of chemotherapy [24]. For instance, Li et al. report that acidic pH impairs ascorbic acid uptake, reducing its cytotoxicity in prostate cancer cells [20]. Proposed mechanisms include selection for apoptosis‐resistant phenotypes [25] and ion gradient effects on drug distribution or ion trapping [26]. Prostate cancer commonly evolves from an androgen‐dependent, AR‐driven state toward castration‐resistant disease, a stage in which AR signaling often remains functionally active despite androgen deprivation [27, 28]. Within the tumor microenvironment, extracellular acidosis can impair the uptake and effectiveness of weakly basic drugs through pH‐dependent drug distribution, thereby reducing their intracellular availability [29–31]. At the same time, acidic conditions have been shown to induce proinvasive phenotypes and epithelial–mesenchymal transition (EMT)‐related remodeling, suggesting that acidosis activates cellular programs that extend beyond altered drug distribution alone [32]. These findings raise the question of whether the reduced efficacy of antiandrogens under acidic conditions mainly reflects AR‐dependent effects on apoptosis and cell cycle regulation, or whether it is driven by AR‐independent adaptive responses associated with EMT and increased migratory capacity [8, 27, 32]. To explore this mechanistic issue across distinct AR contexts, we compare LNCaP and PC‐3 cells under neutral and acidic conditions and examine whether extracellular acidosis differentially influences bicalutamide responsiveness, alongside changes in EMT‐associated traits.

## 2. Method

### 2.1. Cell Lines and Cell Culture

The LNCaP (ATCC Number: CRL‐10995, NCBI Code: C439) and PC3 (ATCC Number: CRL‐1435, NCBI Code: C427) prostate cancer cell lines were prepared from the National Cell Bank of the Pasteur Institute, Tehran, Iran. These cell lines were cultured in Dulbecco’s modified Eagle medium (DMEM, Gibco, Carlsbad, CA) supplemented with 10% heat‐inactivated fetal bovine serum (FBS), 100 units/mL of penicillin, 2 mM L‐glutamine, and 100 μg/mL streptomycin (Gibco BRL, Grand Island, NY). Cultures were maintained in a humidified incubator at 37°C and 5% CO_2_. Experimental control conditions used complete DMEM with 1% dimethyl sulfoxide (DMSO) (Sigma‐Aldrich, St. Louis, MO, USA). A stock solution of bicalutamide (CAS: 90357‐06‐5, Sobhan Oncology) was prepared in DMSO for cell treatments. All experiments for control and treatment groups were conducted at two pH values: 6.8 (acidic) and 7.4 (neutral). Hydrochloric acid (HCl, CAS: 7647‐01‐0, Sigma‐Aldrich) was adjusted to acidic pH. A 25 mM solution of 4‐(2‐hydroxyethyl)‐1‐piperazineethanesulfonic acid (HEPES, CAS: 7365‐45‐9, Sigma‐Aldrich) maintained a constant pH.

### 2.2. Analysis of Cell Survival

The MTT assay (a colorimetric test for cell metabolic activity) was used to evaluate the inhibitory effects of bicalutamide at different pH levels on PC3 and LNCaP prostate cancer cell lines. Cells were seeded at 5 × 10^3^ cells per well in 96‐well plates and incubated for 24 h. Various concentrations of bicalutamide were applied, and cells were incubated at 37°C under 5% CO_2_. For the MTT assay, 5 mg of MTT powder (a yellow tetrazole; Sigma‐Aldrich, St. Louis, MO, USA) was dissolved in 1 mL of sterile phosphate‐buffered saline (PBS) to obtain a 5 mg/mL solution. This was diluted to 10 mL with PBS for a final concentration of 0.5 mg/mL. The solution was freshly prepared for each experiment. After 24 h of treatment, 100 μL of MTT (0.5 mg/mL) was added per well. Plates were incubated for 4 h at 37°C. Formazan crystals (insoluble purple compounds formed by reduction of MTT in living cells) were dissolved using 100 μL of dimethyl acetate (DMA) (Sigma‐Aldrich, St. Louis, MO, USA), producing a purple solution. Absorbance was measured at 545 nm using an ELISA microplate reader (MPR4+, Hiperion, Medizintechnik GmbH & Co.KG, Germany). To determine the half‐maximal inhibitory concentration (IC50) of bicalutamide on PC3 and LNCaP cell lines, assays were conducted in triplicate. Dose–response curves were established and analyzed using GraphPad PRISM software, Version 9 (San Diego, CA), to calculate IC50. Cell viability percentage was calculated from the MTT assay using the following formula:

(1) average absorbance of treated cellsaverage absorbance of control cells×100=% cell  viability.

Absorbance values from triplicate wells were averaged, and cell viability was expressed as a percentage relative to untreated controls.

### 2.3. Measurement of Cell Migration

PC3 and LNCaP cell lines were cultured in six‐well plates at 5 × 10^5^ cells per well. Once the cells reached about 85% confluence, a sterile pipette (P100) tip was used to make a straight wound across the center of the well. After creating the wound, wells were gently washed with PBS to remove detached cells. Cells were treated with bicalutamide at different pH values. Images were taken at wound initiation and after 24 h to track closure. Images were analyzed, and wound‐healing rates were calculated using ImageJ software with the following formula:

(2) A0−AtA0×100=% wound closure,

where *A*_0_ represents the wound area at time 0 and *A*_*t*_ represents the wound area at 24 h.

### 2.4. Measurement of Cell Apoptosis by Flow Cytometry

Apoptosis was evaluated using a fluorescein‐conjugated annexin V (annexin V‐FITC) staining kit as per the manufacturer’s instructions. LNCaP and PC3 cells were cultured in six‐well plates at 5 × 10^5^ cells per well and treated with bicalutamide for 24 h at varying pH values. After incubation, cells were washed twice with PBS and then incubated with a 100 μL mix of annexin V and propidium iodide (PI) for 15 min at room temperature in the dark. Flow cytometry assessed the stained cells. Live cells were in the lower left quadrant (Q4, annexin V‐negative/PI‐negative), early apoptotic cells in the lower right (Q3, annexin V‐positive/PI‐negative), late apoptotic cells in the upper right (Q2, annexin V‐positive/PI‐positive), and necrotic cells in the upper left quadrant (Q1, annexin V‐negative/PI‐positive). Apoptosis rate was determined by the percentage of annexin V‐positive and PI‐negative cells (annexin V+/PI‐), measured with a BD flow cytometer. Data were analyzed using FlowJo software (Tree Star Inc., Version 9.6.3, USA).

### 2.5. DNA Cell Cycle Analysis

Cell cycle analysis was performed using the PI staining technique, which stains DNA and allows cellular DNA content to be measured by fluorescence. PC3 and LNCaP cells, two prostate cancer cell lines, were seeded at 5 × 10^5^ cells per well and exposed to bicalutamide, an AR inhibitor, for 24 h at different pH values. After incubation, cells were washed twice with PBS and then fixed in 70% ethanol at −20°C overnight. The fixed cells were washed again, treated with RNase I (100 μg/mL, which degrades RNA), and stained with 500 μL of PI solution (50 μg/mL in 0.1% Triton X‐100, a detergent that permeabilizes cell membranes, and 0.1% sodium citrate, a buffer) for 30 min at 37°C. The DNA content was analyzed by BD flow cytometry, allowing classification of cells based on the DNA content and identification of apoptotic cells as those in the Sub‐G0/G1 phase (cells with less DNA than those in the G0/G1 phase). Data were analyzed using FlowJo software (Tree Star Inc., Version 9.6.3, USA).

### 2.6. RNA Extraction, cDNA Synthesis, and Gene Expression Analysis by quantitative PCR (qPCR)

Total RNA was extracted with RiboEx (Cat. No. 301‐001/301‐002) reagent following the manufacturer’s instructions. About 1 million cells were mixed with the reagent, incubated, and centrifuged; the supernatant was collected. Chloroform was added, and the mixture was centrifuged again to separate the aqueous phase. Isopropanol precipitated the RNA, which was washed with ethanol to finish the extraction. RNA purity and concentration were measured with a Nanodrop ND‐1000 spectrophotometer (Nanodrop Technologies, Wilmington, DE) at 260 nm and 280 nm. For reverse transcription, 4 μg total RNA was converted to cDNA using the PrimeScript RT reagent Kit (Anacell Co., Iran). The cDNA samples were amplified using a real‐time qPCR cycler (QIAGEN). The B2M gene was used to normalize gene expression, and relative levels were calculated by the 2^−ΔΔCT^ method. Primers and amplicon lengths are listed in Table 1.

**TABLE 1:** Nucleotide sequences of primers used for qPCR.

Gene	Forward primer (5′–3′)	Reverse primer (5′–3′)
E‐cadherin	TCG​TAA​CGA​CGT​TGC​ACC​AA	TTC​GGA​ACC​GCT​TCC​TTC​AT
N‐cadherin	GCC​ATC​AAG​CCT​GTG​GGA​AT	GGA​GCC​ACT​GCC​TTC​ATA​GT
VEGF‐C	GCT​TCT​TCT​CTG​TGG​CGT​GT	CTT​TGC​TTG​CAT​AAG​CCG​TGG
SNAI 1	TAG​CGA​GTG​GTT​CTT​CTG​CG	AGG​GCT​GCT​GGA​AGG​TAA​AC
AR	GACCTTACGGGGACATGC	CGC​ACA​GGT​ACT​TCT​GTT​TCC
BCL2	CCC​CGC​GAC​TCC​TGA​TTC​AT	CAG​TCT​ACT​TCC​TCT​GTG​ATG​TTG​T
BAX	CGG​GTT​GTC​GCC​CTT​TTC​TAC	AGT​CCA​ATG​TCC​AGC​CCA​TGA
B2M	TGT​CTT​TCA​GCA​AGG​ACT​GGT	TGC​TTA​CAT​GTC​TCG​ATC​CCA​C

### 2.7. Statistical Analysis

Data are presented as mean ± standard deviation (SD) from three independent biological experiments. Statistical analysis used one‐way analysis of variance (ANOVA) and *t*‐tests. Differences were considered statistically significant at these levels: ^∗^ *p* < 0.05, ^∗∗^ *p* < 0.01, ^∗∗∗^ *p* < 0.001, and ^∗∗∗∗^ *p* < 0.0001 versus controls.

## 3. Results

### 3.1. Effect of Bicalutamide on Cell Proliferation Under Acidic and Neutral Conditions

The cytotoxicity of bicalutamide in PC3 and LNCaP cell lines was evaluated at acidic and neutral pH. The pH stability was verified after 24 h of incubation under standard culture conditions (Table 2). After 24 h, bicalutamide treatment resulted in higher viability under acidic than under neutral conditions: at 140 μg/mL, PC3 viability was 39.62% (neutral) and 51.36% (acidic), and LNCaP viability was 33.64% (neutral) versus 56.09% (acidic) (*p* < 0.0001 for both; see Figures 1(a) and 1(b)).

**TABLE 2:** The pH of the medium after culturing LNCaP and PC3 cells for 24 h under standard conditions.

Group name	pH after 24 h of incubation
LNCaP—7.4	7.21 ± 0.016
LNCaP—6.8	6.57 ± 0.033
PC3—7.4	7.33 ± 0.049
PC3—6.8	6.77 ± 0.048

*Note:* The initial pH values for the neutral and acidic conditions were 7.41 ± 0.025 and 6.82 ± 0.057, respectively.

FIGURE 1MTT assay of PC3 and LNCaP cells. Effect of extracellular pH on bicalutamide‐induced cytotoxicity in PC3 and LNCaP cells. Cell viability was assessed using the MTT assay after 24‐h treatment with increasing concentrations of bicalutamide under neutral (pH 7.4) and acidic (pH 6.8) conditions. (a) AR‐negative PC3 cells. (b) AR‐positive LNCaP cells. Data are presented as mean ± SD from three independent experiments (*n* = 3). ^∗^ *p* < 0.05, ^∗∗^ *p* < 0.01, ^∗∗∗^ *p* < 0.001, ^∗∗∗∗^ *p* < 0.0001.

**Figure:**
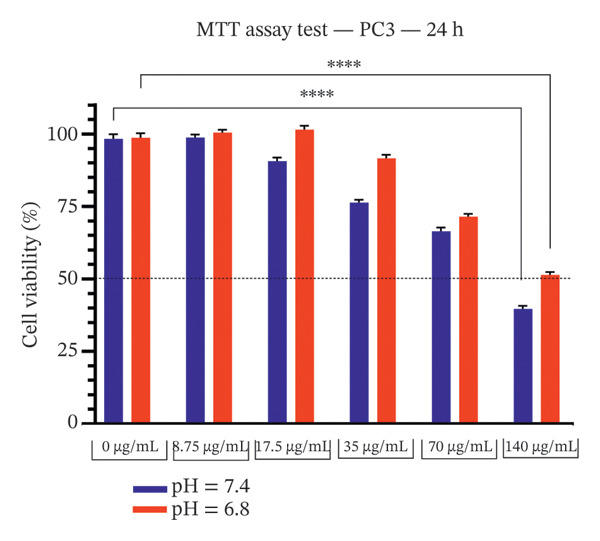
(a)

**Figure:**
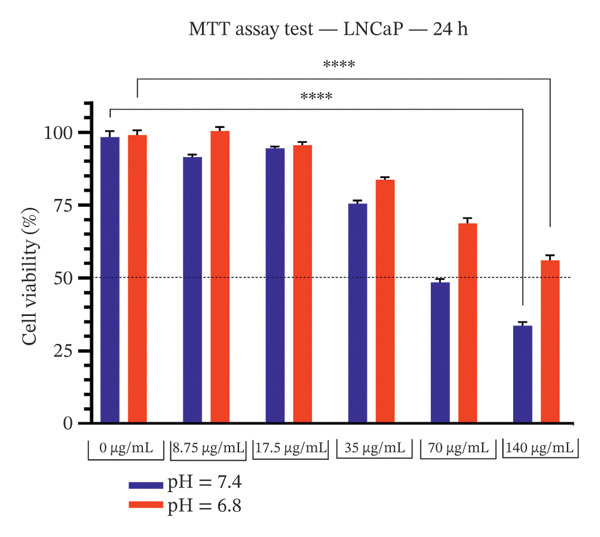
(b)

### 3.2. Role of Bicalutamide in Inhibiting Prostate Cancer Cell Motility Under Different pH Environments

**FIGURE 2:**
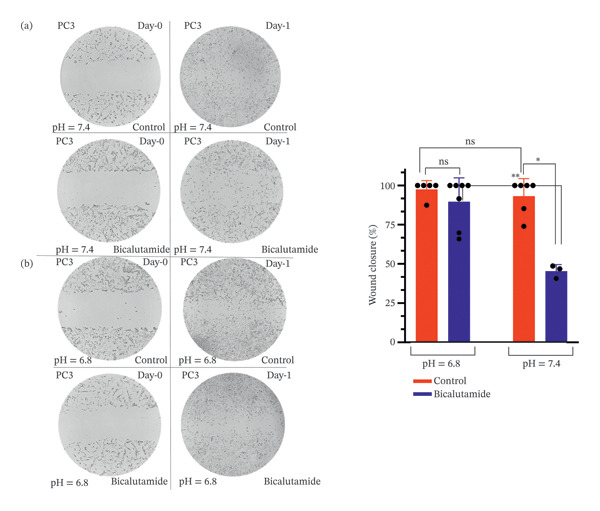
Migration assay of PC3 cells. Cell migration was evaluated using a wound‐healing assay under neutral (pH 7.4) and acidic (pH 6.8) conditions following bicalutamide treatment. (a) Representative quantification of wound closure under neutral conditions. (b) Representative quantification under acidic conditions. Data are presented as mean ± SD from three independent experiments (*n* = 3). ^∗^ *p* < 0.05, ^∗∗^ *p* < 0.01.

Bicalutamide (70 μg/mL for LNCaP, 100 μg/mL for PC3) significantly inhibited migration after 24 h. Under neutral conditions, PC3 migration decreased from 93% (control) to 45% (treated; *p* < 0.05), whereas under acidic conditions, migration remained similar in both groups (97% vs 90%; *p* > 0.05). Treated PC3 cells showed higher migration under acidic (90%) than under neutral (45%) conditions (*p* < 0.01, Figures 2(a) and 2(b)).

**FIGURE 3:**
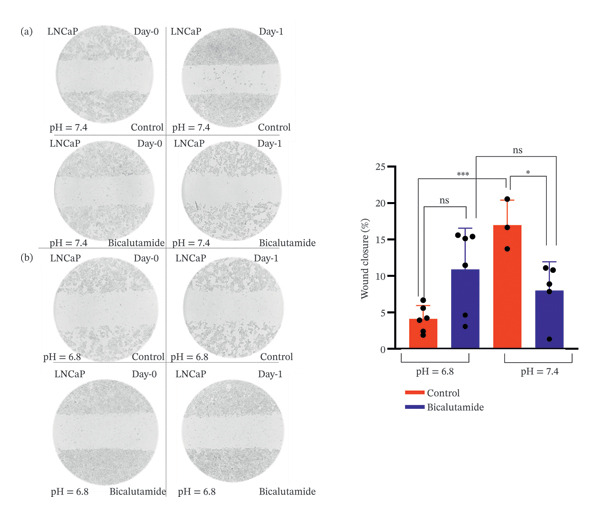
Migration assay of LNCaP cells. Cell migration was assessed using a wound‐healing assay under neutral (pH 7.4) and acidic (pH 6.8) conditions following bicalutamide treatment. (a) Quantification of wound closure under neutral conditions. (b) Quantification under acidic conditions. Data are presented as mean ± SD from three independent experiments (*n* = 3). ^∗^ *p* < 0.05.

A similar trend was observed in LNCaP cells. Under neutral conditions, bicalutamide significantly reduced migration from 17% in the control group to 8% in the treated group (*p* < 0.05). No significant difference was detected between control (4%) and treated (10%) groups under acidic conditions (*p* > 0.05). Additionally, the intrinsic migration capacity of LNCaP cells was significantly higher at neutral pH than at acidic pH (*p* < 0.001; Figures 3(a) and 3(b)).

### 3.3. Impact of Tumor Microenvironment on Bicalutamide‐Mediated Apoptotic Responses

Bicalutamide’s apoptotic effects under acidic and neutral conditions were assessed via flow cytometry. In PC3 cells (140 μg/mL for PC3, 100 μg/mL for LNCaP), neutral pH increased early apoptosis from 5.89% to 12.13% (*p* < 0.05), and late apoptosis from 6.09% to 28.3% (*p* < 0.001). Under acidic pH, early apoptosis remained unchanged, while late apoptosis rose from 9.18% to 31.93% (*p* < 0.001; Figures 4(a) and 4(b)).

FIGURE 4Flow cytometric analysis of apoptosis in PC3 and LNCaP cells. Apoptosis was evaluated by Annexin V/PI staining following bicalutamide treatment under neutral (pH 7.4) and acidic (pH 6.8) conditions. (a and b) Quantification of early and late apoptosis in AR‐negative PC3 cells. (c and d) Quantification of early and late apoptosis in AR‐positive LNCaP cells. Data are presented as mean ± SD from three independent experiments (*n* = 3). ^∗^ *p* < 0.05, ^∗∗^ *p* < 0.01, ^∗∗∗^ *p* < 0.001, ^∗∗∗∗^ *p* < 0.0001.

**Figure:**
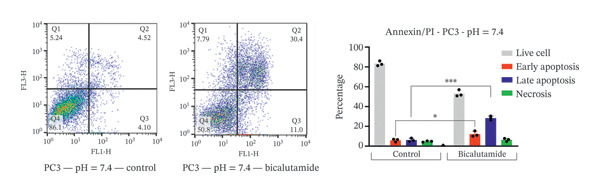
(a)

**Figure:**
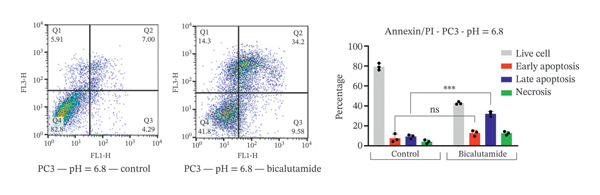
(b)

**Figure:**
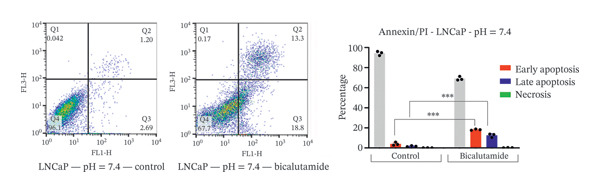
(c)

**Figure:**
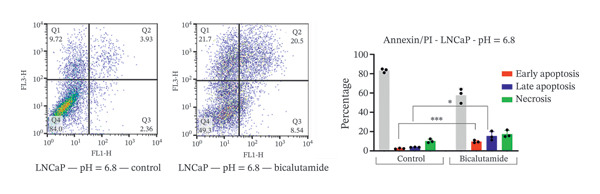
(d)

A similar trend occurred in LNCaP: neutral pH increased early apoptosis from 3.93% to 18.13% and late apoptosis from 1.66% to 12.47% (both *p* < 0.001). Acidic conditions raised early apoptosis from 2.41% to 9.62% (*p* < 0.001) and late from 3.75% to 15.4% (*p* < 0.05; Figures 4(c) and 4(d)).

A comparison of pH conditions showed that in PC3 cells, bicalutamide under neutral conditions reduced early apoptosis from 6.24% to 5.19% (ns). Late apoptosis went from 22.20% to 22.75% (ns). Necrosis increased with acidity, rising from 1.71% at neutral pH to 8.49% at acidic pH (*p* < 0.001, Figure 5(a)).

FIGURE 5Differential apoptotic and necrotic responses to bicalutamide under neutral and acidic conditions. The figure shows the mean percentage differences between bicalutamide‐treated and control cells for early apoptosis, late apoptosis, and necrosis under neutral (pH 7.4) and acidic (pH 6.8) conditions. (a) AR‐negative PC3 cells. (b) AR‐positive LNCaP cells. Blue bars represent pH 7.4, and red bars represent pH 6.8. Each bar corresponds to the mean difference (%) derived from three independent biological replicates (*n* = 3). Statistical comparisons between pH 7.4 and pH 6.8 for each cell death parameter are indicated as follows: ^∗^ *p* < 0.05, ^∗∗^ *p* < 0.01, ^∗∗∗^ *p* < 0.001; ns, not significant.

**Figure:**
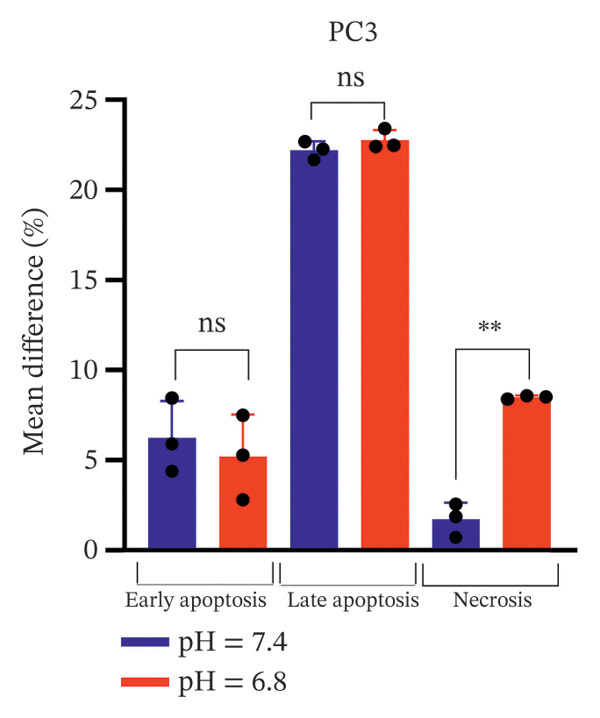
(a)

**Figure:**
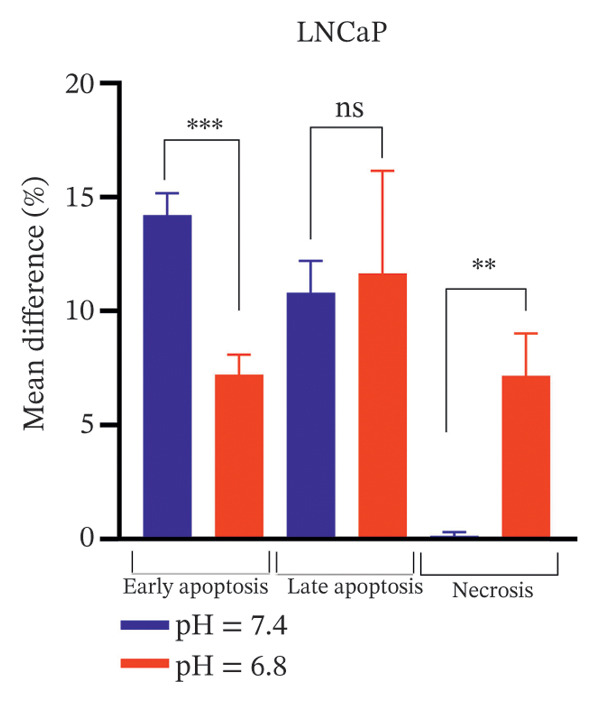
(b)

In LNCaP cells, early apoptosis decreased under acidic conditions, from 14.20% at pH 7.4% to 7.21% at pH 6.8 (*p* < 0.05). Late apoptosis increased slightly, from 10.80% under neutral conditions to 11.65% under acidic conditions (ns). Necrosis also rose under acidic conditions, from 0.147% to 7.15% (*p* < 0.0001, Figure 5(b)).

### 3.4. Influence of pH on Cell Cycle Regulation in Bicalutamide‐Treated Cells

The effects of bicalutamide on cell cycle regulation in prostate cancer cell lines under different pH conditions were evaluated using flow cytometry (see Figure 6). In PC3 metastatic cells treated with bicalutamide (100 μg/mL for LNCaP and 140 μg/mL for PC3), no significant changes were observed in cell cycle distribution under neutral conditions (Figure 6(a)). Under acidic conditions, the G1 phase population decreased from 64.85% to 42.9% (*p* < 0.01, Figure 6(b)). The S phase increased from 26.3% to 40.8% (*p* < 0.05), and the G2/M phase also increased from 8.15% to 16.3% (*p* < 0.05). There were no significant changes in the Sub‐G1 population in PC3 cells under either acidic or neutral conditions after bicalutamide treatment.

FIGURE 6Cell cycle analysis of control and bicalutamide‐treated prostate cancer cells. Cell cycle distribution was analyzed by flow cytometry under neutral (pH 7.4) and acidic (pH 6.8) conditions. (a and b) Quantification of G1, S, and G2/M phase populations in AR‐negative PC3 cells. (c and d) Quantification in AR‐positive LNCaP cells. Data are presented as mean ± SD from three independent experiments (*n* = 3). Statistical comparisons were made between control and bicalutamide‐treated groups at each pH condition. ^∗^ *p* < 0.05, ^∗∗^ *p* < 0.01, ^∗∗∗^ *p* < 0.001, ^∗∗∗∗^ *p* < 0.0001.

**Figure:**
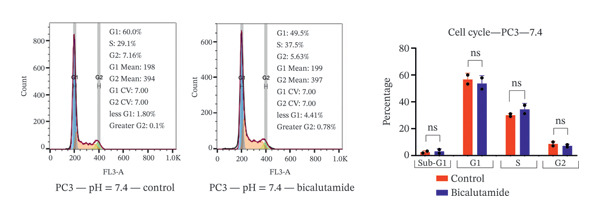
(a)

**Figure:**
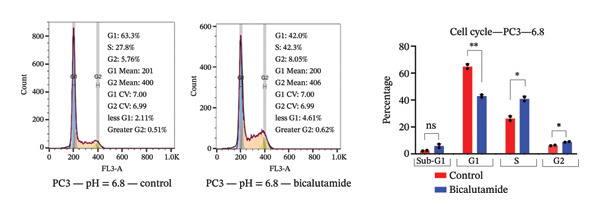
(b)

**Figure:**
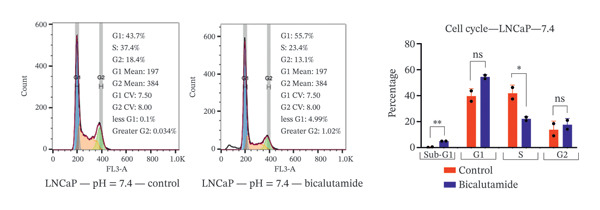
(c)

**Figure:**
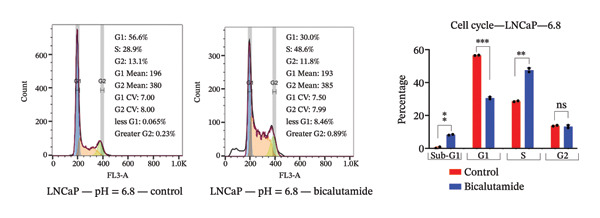
(d)

In LNCaP cells, bicalutamide treatment under neutral conditions increased the Sub‐G1 population from 0.3% to 4.96% (*p* < 0.01), while the S phase decreased from 41.85% to 22.1% (*p* < 0.05, Figure 6(c)). Under acidic conditions, both the Sub‐G1 population (from 0.49% to 8.23%, *p* < 0.01) and the S phase (from 28.65% to 47.55%, *p* < 0.01) increased. The G1 phase population decreased from 56.5% to 30.6% (*p* < 0.001, Figure 6(d)).

A comparison of cell cycle distribution showed that the Sub‐G1 population in PC3 cells did not change much after bicalutamide treatment, whether under neutral or acidic conditions (mean differences: 0.615% at pH 7.4 and 3.51% at pH 6.8, *p* > 0.05). However, the S and G2/M phases increased more under acidic conditions, with mean differences of 14.75% (*p* < 0.05, S phase) and 2.65% (*p* < 0.05, G2/M) at pH 6.8, compared to about 4.3% (S phase) and −1.48% (G2/M) at pH 7.4. The G1 phase, on the other hand, dropped sharply under acidic conditions, falling to −21.95% compared to −3% under neutral conditions (*p* < 0.01, Figure 7(a)).

FIGURE 7Differential cell cycle phase distribution between bicalutamide‐treated and control cells under neutral and acidic conditions. The figure illustrates the mean percentage differences between bicalutamide‐treated and control cells in the Sub‐G1, G1, S, and G2 phases under neutral (pH 7.4) and acidic (pH 6.8) conditions. (a) AR‐negative PC3 cells. (b) AR‐positive LNCaP cells. Blue bars represent pH 7.4, and red bars represent pH 6.8. Each bar corresponds to the mean difference (%) derived from three independent biological replicates (*n* = 3). Statistical comparisons between pH 7.4 and pH 6.8 for each cell cycle phase are indicated as follows: ^∗^ *p* < 0.05, ^∗∗^ *p* < 0.01; ns, not significant.

**Figure:**
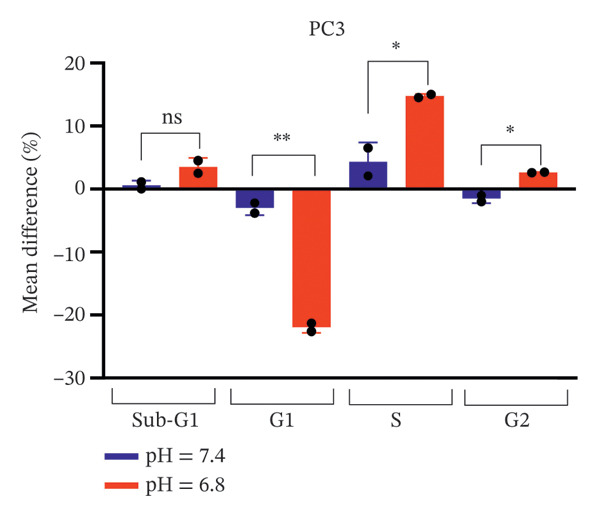
(a)

**Figure:**
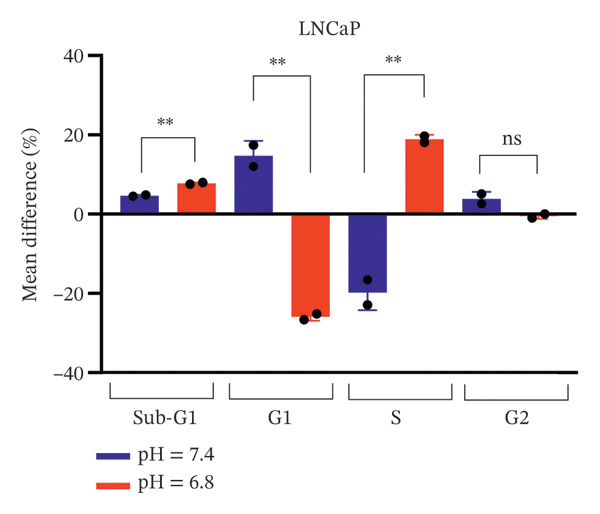
(b)

In LNCaP cells, the Sub‐G1 population increased under acidic conditions, going from a mean difference of 4.66% at pH 7.4 to 7.74% at pH 6.8 (*p* < 0.01). The G1 phase dropped sharply under acidic conditions, from 14.7% at neutral pH to −25.9% at acidic pH (*p* < 0.01). The S phase population rose significantly at pH 6.8, with a mean difference of 18.9%, compared to −19.75% at pH 7.4 (*p* < 0.01). The G2/M population decreased slightly under acidic conditions, from 3.85% at pH 7.4 to −0.5% at pH 6.8 (Figure 7(b)). These results show that LNCaP cells have greater pH‐dependent changes than PC3 cells, especially in the G1 and S phases, highlighting the important effect of extracellular acidity on bicalutamide‐induced cell cycle changes.

### 3.5. Assessing pH Effects on qPCR Outcomes in Cells Exposed to Bicalutamide

The expression levels of apoptosis‐related genes (*BAX*, *BCL2*), the lymphangiogenesis‐associated gene (VEGF‐C), EMT‐related genes (*SNAI 1*, *E-cadherin*, and *N-cadherin*), and the AR gene were analyzed in PC3 and LNCaP prostate cancer cell lines exposed to bicalutamide (100 μg/mL for LNCaP and 140 μg/mL for PC3 cells) under different pH conditions using qPCR (Figure 8). The relative expression levels of all genes in the treated groups and the control group under acidic conditions were normalized to the control group under neutral conditions, which was set as the reference (equal to 1).

FIGURE 8qPCR analysis of gene expression. Gene expression levels of *BAX*, *BCL2*, *VEGF-C*, *SNAI1*, *E-cadherin*, *N-cadherin*, and *AR* were quantified by qPCR under neutral (pH 7.4) and acidic (pH 6.8) conditions. Expression levels were normalized to the neutral control group, which was set to 1. (a) AR‐negative PC3 cells. (b) AR‐positive LNCaP cells. Data are presented as mean ± SD from three independent experiments (*n* = 3). Statistical significance is indicated as follows: ^∗^ *p* < 0.05, ^∗∗^ *p* < 0.01, ^∗∗∗^ *p* < 0.001, ^∗∗∗∗^ *p* < 0.0001.

**Figure:**
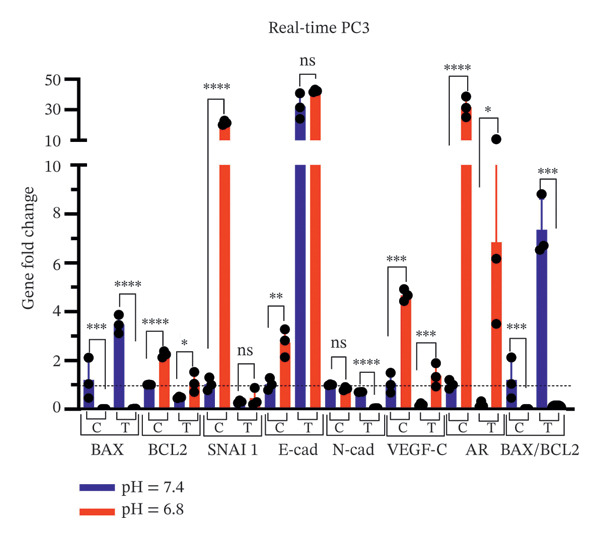
(a)

**Figure:**
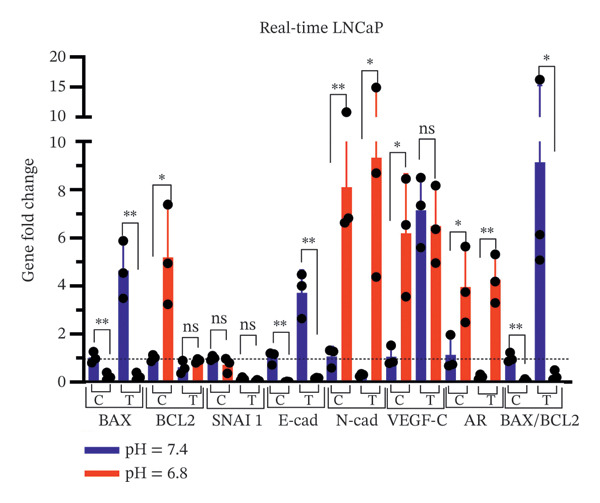
(b)

In PC3 cells, significant changes in gene expression were observed under acidic conditions compared to neutral conditions. Specifically, *BCL2* expression increased significantly in both the control (*p* < 0.0001) and treated groups (*p* < 0.05), while *SNAI 1* in the control group (*p* < 0.0001), *E-cadherin* in the control group (*p* < 0.01), *VEGF-C* (*p* < 0.001 for both control and treated groups), and *AR* (*p* < 0.0001 in the control group and *p* < 0.05 in the treated group) were also significantly upregulated. Conversely, *BAX* expression was significantly downregulated in both the control (*p* < 0.001) and treated groups (*p* < 0.0001). Furthermore, *N-cadherin* expression was significantly decreased in the treated group (*p* < 0.0001, Figure 8(a)).

Similarly, in LNCaP cells, acidic conditions resulted in a significant increase in BCL2 expression in the control group (*p* < 0.05), *N-cadherin* expression in both control (*p* < 0.01) and treated groups (*p* < 0.05), *VEGF-C* expression in the control group (*p* < 0.05), and *AR* expression in both groups (*p* < 0.05 and *p* < 0.01 in the control and treated groups, respectively). In contrast, *BAX* expression was significantly downregulated in both control (*p* < 0.01) and treated groups (*p* < 0.01), as was *E-cadherin* in both groups (*p* < 0.01, Figure 8(b)).

Overall, the results demonstrated that the *BAX/BCL2* ratio, a marker of apoptosis, was significantly reduced under acidic conditions, indicating the suppression of proapoptotic signaling. Additionally, EMT pathway‐related genes were significantly upregulated under acidic conditions, indicating enhanced EMT activity. *VEGF-C* and *AR* expression were also markedly increased under acidic conditions, suggesting that the acidic environment promotes lymphangiogenesis and *AR* signaling. These findings highlight the role of pH in modulating cancer progression pathways, particularly apoptosis, EMT, lymphangiogenesis, and AR‐related mechanisms.

## 4. Discussion

This study offers an initial evaluation of pH’s influence on the efficacy of androgen deprivation in prostate cancer cells. Since solid tumors such as prostate cancer commonly exhibit a decreased extracellular pH (around 6.5–6.9), pH 6.8 was selected to mimic tumor‐associated acidosis, and pH 7.4 was used to reflect the physiological condition. The findings reveal that bicalutamide’s cytotoxic, antimigratory, and proapoptotic effects on PC3 and LNCaP prostate cancer cell lines are significantly more pronounced at neutral pH than under acidic environments. Acidic pH diminishes bicalutamide’s ability to inhibit proliferation and migration, while also differentially impacting cell cycle distribution in the two cell lines. Gene expression analysis of apoptosis‐related factors reveals that acidic conditions suppress proapoptotic signaling, evidenced by a decreased *BAX/BCL2* ratio. Furthermore, acidic environments promote EMT [32, 33]. While AR signaling is also upregulated in acidic pH, the combined effects of these gene expression changes ultimately dominate AR modulation, collectively contributing to a tumor‐supportive microenvironment. These results underscore the pivotal role of extracellular pH in modulating the responsiveness of prostate cancer cells to bicalutamide and in influencing key cancer progression pathways, including apoptosis, motility, and EMT. In addition to using nanoparticles, which have demonstrated improved ADT efficacy in prostate cancer [34], pH modulation of the tumor microenvironment represents another potentially effective strategy. Employing alkalizing agents to elevate extracellular pH could shift the tumor microenvironment toward a more neutral state, potentially augmenting the therapeutic effectiveness of bicalutamide. This approach may mitigate the protective, tumor‐promoting effects of acidity and improve apoptotic responses to treatment.

PC3 cells migrated more at acidic pH than at neutral pH. In contrast, LNCaP cells without bicalutamide moved more at neutral pH than under acidic conditions. LNCaP cells also showed a stronger EMT, with lower E‐cadherin and higher N‐cadherin levels. Acidic stress reduced early apoptosis in LNCaP cells but did not affect PC3 cells. Acidic pH blocked bicalutamide‐induced G1 arrest in LNCaP cells, leading to more cells entering the S phase. In PC3 cells, acidic pH gradually increased S phase cells but did not change the drug’s effect on the cell cycle. Acidic pH altered AR‐dependent survival and antiapoptotic responses in both cell types, but PC3 cells showed a greater increase in *BCL2*, *VEGF-C*, and *AR*. Huang et al. investigated the impact of an acidic extracellular environment on prostate cancer bone metastasis using the PC3 cell line [35]. Their study revealed that an acidic extracellular pH (pHe) enhanced both PC3 cell proliferation and tumor sphere formation. Additionally, this acidic environment increased the expression of stemness‐related markers, such as CD133, CD44, Oct4, and Klf4, in these cells. pHe enhances the stem cell‐like properties of PC3 cells, which are crucial drivers of tumor progression and metastasis [36]. In addition, they have shown that an acidic extracellular microenvironment could facilitate the bone metastasis of prostate cancer by boosting the characteristics of cancer stem cells, which aligns with our observations [35].

Robey et al. showed that oral bicarbonate therapy significantly reduced the incidence of metastases in experimental models of breast and prostate cancer. It is not known whether bicarbonate exerts its effects by decreasing the survival of circulating tumor cells or by inhibiting colonization at the metastatic site, on the one hand, and the acidic pH of the primary tumor may induce a stress response in these cells, leading to increased survival. This would be consistent with previous observations by Hill and colleagues, who showed that the pretreatment of melanoma cells with acid pH before injection enhances survival at metastatic sites [37, 38]. Alternatively, bicarbonate buffering may inhibit local invasion at the metastatic site. This has been formulated as the acid‐mediated invasion hypothesis, in which tumor‐derived acid is secreted into the surrounding parenchyma, leading to degradation of the extracellular matrix [39]. Whether at the primary or metastatic site, acidic pH appears to stimulate invasive behavior and increased survival, either by selection or induction [40–42].

The *VEGF/VEGFR* signaling pathway is crucial for pathological angiogenesis, and antiangiogenic drugs targeting this pathway are being developed to combat cancer [43, 44]. Research indicates that bone marrow‐derived endothelial progenitor cells (BM‐EPCs) express *VEGFR1* and *VEGFR2* [45], with circulating *VEGFR2+* BM‐EPCs correlating with tumor metastasis [46]. *VEGFR2* activates downstream signaling pathways, including Akt and p38, which promote endothelial cell growth, migration, and tube formation [47, 48]. Huang et al. reported that the PC3‐conditioned medium increases the phosphorylation of *VEGFR2*, Akt, and p38 in BM‐EPCs, with this phosphorylation being more pronounced when PC3 cells are cultured at pH 6.5 [35]. The acidic conditioned medium promotes VEGF‐induced vasculogenesis in BM‐EPCs by activating *VEGFR-2*, Akt, and p38 phosphorylation. The PC3‐conditioned medium enhances BM‐EPC proliferation and migration more effectively than normal conditioned medium, with these effects amplified when PC3 cells are cultured at pH 6.5 [35].

El Kenawi et al. propose that acidic pH influences prostate cancer cells through immunological pathways. Specifically, an acidic environment alters the activation state of macrophages under polarizing conditions, pushing them toward a protumor phenotype akin to that of tumor‐associated macrophages (TAMs). Additionally, they found that neutralizing tumor acidity alters TAM activation, leading to a notable decrease in the expression of genes such as Arg1 and Cd206, which are typically linked to tumor‐promoting functions. These findings indicate that tumor acidosis plays a crucial role in shaping macrophage tumor‐supportive behavior in prostate cancer [49].

This study marks the first evaluation of pH’s influence on bicalutamide responsiveness in prostate cancer cells, though the impact of extracellular pH on chemotherapeutic drugs has been previously investigated. Sauvant et al. examined how an acidic environment affects p‐glycoprotein (pGP) activity, cellular content, and cytotoxicity of the chemotherapeutic drug daunorubicin in the AT1 R‐3327 Dunning prostate carcinoma cell line, both in vitro and in vivo. Their in vitro findings showed that extracellular acidosis (pH 6.6) activated p38 and ERK1/2, leading to daunorubicin resistance through significant pGP activation. This activation did not require new protein synthesis, and transport kinetics analysis revealed rapid and sustained pGP activation at pH 6.6 compared to pH 7.4. Intracellular acidification also induced daunorubicin resistance by activating pGP, mediated solely by p38 activation. In vivo, a reduced extracellular pH of 6.6 similarly induced daunorubicin resistance, which could be reversed by inhibiting p38 [50].

Lee et al. evaluated the effect of pHe on the radiation therapy of Mammary adenocarcinoma cells of A/J mice (SCK cells). Radiation‐induced apoptosis declined as the medium pH was lowered from 7.5 to 6.4. Specifically, radiation‐induced DNA degradation, including early DNA breaks, as determined by the TUNEL method, progressively declined as the medium pH was lowered, so that little DNA fragmentation occurred 48 h after irradiation with 12 Gy in pH 6.6 medium. When the cells were irradiated and incubated for 48 h in a pH 6.6 medium, followed by replacement with a pH 7.5 medium, DNA fragmentation promptly occurred. Radiation‐induced G2 arrest is prolonged under an acidic environment, indicating that the suppression of radiation‐induced apoptosis and prolongation of radiation‐induced G2 arrest under an acidic environment are related [51].

The impact of microenvironmental pH on other cancer cells (such as gastric cancer [52] and melanoma [41]) has been investigated. Li et al. examined the effect of extracellular pH on gastric cancer cell lines. MTT assays indicated that the viability of SGC‐7901 and MKN45 cells was significantly lower at pH 8.0 than at pH 6.0 or 7.0 (*p* < 0.001). Flow cytometry revealed that apoptosis was more pronounced at pH 8.0 than at the other pH levels (*p* < 0.001). Transwell assays showed that the invasive ability of these cells was markedly reduced at pH 8.0 compared with pH 6.0 and 7.0 (*p* < 0.001). qPCR and Western blot analyses indicated that increasing pH led to the downregulation of mTOR, AKT, Wnt, Glut, and HIF‐1*α* in both cell lines (*p* < 0.05). These findings align with our results, confirming that cancer cell invasiveness is enhanced under acidic conditions [52].

It is important to acknowledge certain methodological constraints that may affect how the findings are interpreted. First, Western blot analysis was not employed in these experiments, which limited the validation of observed transcriptional changes at the protein level. Future studies should incorporate protein‐level validation using Western blotting to assess the expression of key markers such as *E-cadherin*, *AR*, and *VEGF-C*. This approach would enhance mechanistic insights. Second, our research was conducted exclusively in vitro. Thus, caution should be exercised when extrapolating these results to a physiological context, given the absence of in vivo tests. Third, we examined only two pH levels, 7.4 and 6.8. This choice may not fully capture the pH range in the tumor microenvironment. Additionally, it was not investigated whether the changes induced by acidic pH are reversible. Specifically, future studies should include experiments to test whether neutralizing an acidic environment could reverse its negative effects and explore the reversibility of these processes more broadly. Finally, the molecular mechanisms by which acidic pH exerts its effects, including the signaling pathways involved, remain unclear and warrant further investigation.

## 5. Conclusion

This study concludes that the efficacy of ADT with bicalutamide in prostate cancer cells is significantly influenced by extracellular pH. Specifically, bicalutamide demonstrates enhanced cytotoxic, antimigratory, and proapoptotic effects under neutral pH (7.4) compared to acidic conditions (6.8). Acidic pH diminishes the drug’s effectiveness by reducing its ability to inhibit proliferation and migration, suppressing proapoptotic signaling, promoting EMT, and enhancing both lymphangiogenesis and AR signaling. Upon further in vivo and pharmacodynamic validation of these findings, strategies aimed at modulating the tumor microenvironment pH, such as the use of alkalizing agents, may represent a promising approach to enhance the efficacy of ADT and improve treatment outcomes in prostate cancer.

## Funding

No funding was received for this manuscript.

## Conflicts of Interest

The authors declare no conflicts of interest.

## Data Availability

The data that support the findings of this study are available from the corresponding author upon reasonable request.

## References

[1] Bray F. , Laversanne M. , Sung H. et al., Global Cancer Statistics 2022: GLOBOCAN Estimates of Incidence and Mortality Worldwide for 36 Cancers in 185 Countries, CA: A Cancer Journal for Clinicians. (2024) 74, no. 3, 229–263, 10.3322/caac.21834. 38572751

[2] Chukhu M. , Dahiya U. R. , and Heemers H. V. , Evolving Roles for the Androgen Receptor and Its Protein Interactome in castration-resistant Prostate Cancer, Oncogene. (2025) 44, no. 41, 1–12, 10.1038/s41388-025-03573-z. 40968254 PMC12500472

[3] Damber J. E. and Aus G. , Prostate Cancer, Prostate cancer. Lancet.(2008) 371, no. 9625, 1710–1721, 10.1016/s0140-6736(08)60729-1, 2-s2.0-43449127502. 18486743

[4] Chen Y. , Zhou Q. , Hankey W. , Fang X. , and Yuan F. , Second Generation Androgen Receptor Antagonists and Challenges in Prostate Cancer Treatment, Cell Death & Disease. (2022) 13, no. 7, 10.1038/s41419-022-05084-1. PMC9304354 35864113

[5] Safi R. , Wardell S. E. , Watkinson P. et al., Androgen Receptor Monomers and Dimers Regulate Opposing Biological Processes in Prostate Cancer Cells, Nature Communications. (2024) 15, no. 1, 10.1038/s41467-024-52032-y. PMC11371910 39227594

[6] Moul J. W. , Biochemical Recurrence of Prostate Cancer, Current Problems in Cancer. (2003) 27, no. 5, 243–272, 10.1016/s0147-0272(03)00032-1. 12963877

[7] Hsieh A. C. and Ryan C. J. , Novel Concepts in Androgen Receptor Blockade, The Cancer Journal. (2008) 14, no. 1, 11–14, 10.1097/ppo.0b013e318161d13e, 2-s2.0-43049167422. 18303477

[8] Karantanos T. , Corn P. G. , and Thompson T. C. , Prostate Cancer Progression After Androgen Deprivation Therapy: Mechanisms of Castrate Resistance and Novel Therapeutic Approaches, Oncogene. (2013) 32, no. 49, 5501–5511, 10.1038/onc.2013.206, 2-s2.0-84890555121. 23752182 PMC3908870

[9] Shand R. L. and Gelmann E. P. , Molecular Biology of prostate-cancer Pathogenesis, Current Opinion in Urology. (2006) 16, no. 3, 123–131, 10.1097/01.mou.0000193384.39351.64, 2-s2.0-33646940750. 16679847

[10] Kageyama Y. , Hyochi N. , Kihara K. , and Sugiyama H. , The Androgen Receptor as Putative Therapeutic Target in hormone-refractory Prostate Cancer, Frontiers in Anti-Cancer Drug Discovery. (2010) 1, 481–496. 10.2174/157489207782497172 18221063

[11] Schrijvers D. , Androgen-Independent Prostate Cancer, Prostate Cancer.(2007) 239–249. 10.1007/978-3-540-40901-4_14 17432563

[12] Galletti G. , Leach B. I. , Lam L. , and Tagawa S. T. , Mechanisms of Resistance to Systemic Therapy in Metastatic castration-resistant Prostate Cancer, Cancer Treatment Reviews. (2017) 57, 16–27, 10.1016/j.ctrv.2017.04.008, 2-s2.0-85019477883. 28527407

[13] Konerding M. , Malkusch W. , Klapthor B. et al., Evidence for Characteristic Vascular Patterns in Solid Tumours: Quantitative Studies Using Corrosion Casts, British Journal of Cancer. (1999) 80, no. 5, 724–732, 10.1038/sj.bjc.6690416, 2-s2.0-0032952966. 10360650 PMC2362271

[14] Vaupel P. , Oxygen Supply to Malignant Tumors, Tumor Blood Circulation. (2020) CRC Press, 143–168.

[15] Hockel M. and Vaupel P. , Tumor Hypoxia: Definitions and Current Clinical, Biologic, and Molecular Aspects, Journal of the National Cancer Institute. (2001) 93, no. 4, 266–276, 10.1093/jnci/93.4.266, 2-s2.0-0035925098. 11181773

[16] Rolver M. G. , Holland L. K. , Ponniah M. et al., Chronic Acidosis Rewires Cancer Cell Metabolism Through Pparα Signaling, International Journal of Cancer. (2023) 152, no. 8, 1668–1684, 10.1002/ijc.34404. 36533672 PMC10108231

[17] Galapate C. M. and Commisso C. , Organellar Ph as an Emerging Vulnerability to Exploit in Cancer, Trends in Cancer. (2025) . 10.1016/j.trecan.2025.09.006 PMC12616702 41136318

[18] Flavell R. R. , Truillet C. , Regan M. K. et al., Caged [18F] FDG Glycosylamines for Imaging Acidic Tumor Microenvironments Using Positron Emission Tomography, Bioconjugate Chemistry. (2016) 27, no. 1, 170–178, 10.1021/acs.bioconjchem.5b00584, 2-s2.0-84955593102. 26649808 PMC4854293

[19] Anderson M. , Moshnikova A. , Engelman D. M. , Reshetnyak Y. K. , and Andreev O. A. , Probe for the Measurement of Cell Surface Ph in Vivo and Ex Vivo, Proceedings of the National Academy of Sciences. (2016) 113, no. 29, 8177–8181, 10.1073/pnas.1608247113, 2-s2.0-84978835850. PMC4961157 27382181

[20] Li Z. , He P. , Luo G. et al., Increased Tumoral Microenvironmental pH Improves Cytotoxic Effect of Pharmacologic Ascorbic Acid in castration-resistant Prostate Cancer Cells, Frontiers in Pharmacology. (2020) 11, 10.3389/fphar.2020.570939. PMC7538777 33071784

[21] Raghunand N. , He X. , Van Sluis R. et al., Enhancement of Chemotherapy by Manipulation of Tumour pH, British Journal of Cancer. (1999) 80, no. 7, 1005–1011, 10.1038/sj.bjc.6690455, 2-s2.0-0032904346. 10362108 PMC2363059

[22] Esmaeilzadeh S. , Valizadeh H. , and Zakeri-Milani P. , The Effects of pH, Temperature and Protein Concentration on the in Vitro Binding of Flutamide to Human Serum Albumin, Pharmaceutical Development and Technology. (2017) 22, no. 8, 982–991, 10.3109/10837450.2016.1163392, 2-s2.0-84964001357. 27055586

[23] Saroj S. and Rajput S. J. , Facile Development, Characterization, and Evaluation of Novel Bicalutamide Loaded pH-sensitive Mesoporous Silica Nanoparticles for Enhanced Prostate Cancer Therapy, Drug Development and Industrial Pharmacy. (2019) 45, no. 4, 532–547, 10.1080/03639045.2018.1562463, 2-s2.0-85060923802. 30582382

[24] Mahoney B. P. , Raghunand N. , Baggett B. , and Gillies R. J. , Tumor Acidity, Ion Trapping and Chemotherapeutics: I. Acid pH Affects the Distribution of Chemotherapeutic Agents in Vitro, Biochemical Pharmacology. (2003) 66, no. 7, 1207–1218, 10.1016/s0006-2952(03)00467-2, 2-s2.0-0141786825. 14505800

[25] Ohtsubo T. , Igawa H. , Saito T. et al., Acidic Environment Modifies heat-or radiation-induced Apoptosis in Human Maxillary Cancer Cells, International Journal of Radiation Oncology, Biology, Physics. (2001) 49, no. 5, 1391–1398, 10.1016/s0360-3016(00)01590-x, 2-s2.0-0035312445. 11286847

[26] Raghunand N. and Gillies R. J. , pH and Drug Resistance in Tumors, Drug Resistance Updates. (2000) 3, no. 1, 39–47, 10.1054/drup.2000.0119, 2-s2.0-0034143570. 11498364

[27] Coutinho I. , Day T. K. , Tilley W. D. , and Selth L. A. , Androgen Receptor Signaling in castration-resistant Prostate Cancer: a Lesson in Persistence, Endocrine-Related Cancer. (2016) 23, no. 12, T179–T197, 10.1530/erc-16-0422, 2-s2.0-84997719771. 27799360

[28] Katzenwadel A. and Wolf P. , Androgen Deprivation of Prostate Cancer: Leading to a Therapeutic Dead End, Cancer Letters. (2015) 367, no. 1, 12–17, 10.1016/j.canlet.2015.06.021, 2-s2.0-84939267751. 26185001

[29] Wojtkowiak J. W. , Verduzco D. , Schramm K. J. , and Gillies R. J. , Drug Resistance and Cellular Adaptation to Tumor Acidic pH Microenvironment, Molecular Pharmaceutics. (2011) 8, no. 6, 2032–2038, 10.1021/mp200292c, 2-s2.0-82955173044. 21981633 PMC3230683

[30] Swietach P. , Hulikova A. , Patiar S. , Vaughan-Jones R. D. , and Harris A. L. , Importance of Intracellular pH in Determining the Uptake and Efficacy of the Weakly Basic Chemotherapeutic Drug, Doxorubicin, PLoS One. (2012) 7, no. 4, 10.1371/journal.pone.0035949, 2-s2.0-84860372850. PMC3338554 22563426

[31] Gerweck L. E. , Vijayappa S. , and Kozin S. , Tumor pH Controls the in Vivo Efficacy of Weak Acid and Base Chemotherapeutics, Molecular Cancer Therapeutics. (2006) 5, no. 5, 1275–1279, 10.1158/1535-7163.mct-06-0024, 2-s2.0-33745110220. 16731760

[32] Corbet C. , Bastien E. , Santiago de Jesus J. P. et al., TGFβ2-induced Formation of Lipid Droplets Supports acidosis-driven EMT and the Metastatic Spreading of Cancer Cells, Nature Communications. (2020) 11, no. 1, 10.1038/s41467-019-14262-3. PMC6978517 31974393

[33] Zhu S. , Zhou H.-Y. , Deng S.-C. et al., ASIC1 and ASIC3 Contribute to acidity-induced EMT of Pancreatic Cancer Through Activating Ca2+/RhoA Pathway, Cell Death & Disease. (2017) 8, no. 5, e2806–e, 10.1038/cddis.2017.189, 2-s2.0-85032005436. 28518134 PMC5520710

[34] Zareian Baghdadabad L. , Menbari Oskouie I. , Yahyazadeh S. R. et al., Improved Anticancer Properties of Silver Nanoparticles by Albumin Coating in Prostate Cancer Cell Lines: an in Vitro Study, Pharmaceutics. (2026) 18, no. 3, 10.3390/pharmaceutics18030338. PMC13028938 41900824

[35] Huang S. , Tang Y. , Peng X. et al., Acidic Extracellular pH Promotes Prostate Cancer Bone Metastasis by Enhancing PC-3 Stem Cell Characteristics, Cell Invasiveness and VEGF-Induced Vasculogenesis of BM-EPCs, Oncology Reports. (2016) 36, no. 4, 2025–2032, 10.3892/or.2016.4997, 2-s2.0-84984985687. 27498716

[36] Michl M. , Heinemann V. , Jung A. , Engel J. , Kirchner T. , and Neumann J. , Expression of Cancer Stem Cell Markers in Metastatic Colorectal Cancer Correlates with Liver Metastasis, but Not with Metastasis to the Central Nervous System, Pathology, Research & Practice. (2015) 211, no. 8, 601–609, 10.1016/j.prp.2015.05.006, 2-s2.0-84983094727. 26092596

[37] Rofstad E. K. , Mathiesen B. , Kindem K. , and Galappathi K. , Acidic Extracellular pH Promotes Experimental Metastasis of Human Melanoma Cells in Athymic Nude Mice, Cancer Research. (2006) 66, no. 13, 6699–6707, 10.1158/0008-5472.can-06-0983, 2-s2.0-33746149756. 16818644

[38] Schlappack O. , Zimmermann A. , and Hill R. , Glucose Starvation and Acidosis: Effect on Experimental Metastatic Potential, DNA Content and MTX Resistance of Murine Tumour Cells, British Journal of Cancer. (1991) 64, no. 4, 663–670, 10.1038/bjc.1991.378, 2-s2.0-0025771024. 1911214 PMC1977701

[39] Gatenby R. A. , Gawlinski E. T. , Gmitro A. F. , Kaylor B. , and Gillies R. J. , Acid-Mediated Tumor Invasion: a Multidisciplinary Study, Cancer Research. (2006) 66, no. 10, 5216–5223, 10.1158/0008-5472.can-05-4193, 2-s2.0-33744942376. 16707446

[40] Glunde K. , Guggino S. E. , Solaiyappan M. , Pathak A. P. , Ichikawa Y. , and Bhujwalla Z. M. , Extracellular Acidification Alters Lysosomal Trafficking in Human Breast Cancer Cells, Neoplasia. (2003) 5, no. 6, 533–545, 10.1016/s1476-5586(03)80037-4. 14965446 PMC1502575

[41] Martínez-Zaguilán R. , Seftor E. A. , Seftor R. E. , Chu Y.-W. , Gillies R. J. , and Hendrix M. J. , Acidic pH Enhances the Invasive Behavior of Human Melanoma Cells, Clinical & Experimental Metastasis. (1996) 14, 176–186. 8605731 10.1007/BF00121214

[42] Estrella V. , Chen T. , Lloyd M. et al., Acidity Generated by the Tumor Microenvironment Drives Local Invasion, Cancer Research. (2013) 73, no. 5, 1524–1535, 10.1158/0008-5472.can-12-2796, 2-s2.0-84874886049. 23288510 PMC3594450

[43] Lee S. H. , Jeong D. , Han Y.-S. , and Baek M. J. , Pivotal Role of Vascular Endothelial Growth Factor Pathway in Tumor Angiogenesis, Annals of surgical treatment and research. (2015) 89, no. 1, 10.4174/astr.2015.89.1.1, 2-s2.0-84981747169. PMC4481026 26131438

[44] Carmeliet P. , Angiogenesis in Life, Disease and Medicine, Nature. (2005) 438, no. 7070, 932–936, 10.1038/nature04478, 2-s2.0-30744479430. 16355210

[45] Hoffmann B. R. , Wagner J. R. , Prisco A. R. , Janiak A. , and Greene A. S. , Vascular Endothelial Growth factor-A Signaling in Bone marrow-derived Endothelial Progenitor Cells Exposed to Hypoxic Stress, Physiological Genomics. (2013) 45, no. 21, 1021–1034, 10.1152/physiolgenomics.00070.2013, 2-s2.0-84887033837. 24022223 PMC3841787

[46] Fo F. , Gross-Goupil M. , Tournay E. et al., Levels of Circulating CD45dimCD34+ VEGFR2+ Progenitor Cells Correlate with Outcome in Metastatic Renal Cell Carcinoma Patients Treated with Tyrosine Kinase Inhibitors, British Journal of Cancer. (2011) 104, no. 7, 1144–1150. 21386843 10.1038/bjc.2011.72 PMC3068506

[47] Kowshik J. , Giri H. , Kranthi Kiran Kishore T. et al., Ellagic Acid Inhibits VEGF/VEGFR2, PI3K/Akt and MAPK Signaling Cascades in the Hamster Cheek Pouch Carcinogenesis Model, Anti-Cancer Agents in Medicinal Chemistry. (2014) 14, no. 9, 1249–1260, 10.2174/1871520614666140723114217. 25060902

[48] Koch S. and Claesson-Welsh L. , Signal Transduction by Vascular Endothelial Growth Factor Receptors, Cold Spring Harbor Perspectives in Medicine. (2012) 2, no. 7, 10.1101/cshperspect.a006502, 2-s2.0-84880742193. PMC3385940 22762016

[49] El-Kenawi A. , Gatenbee C. , Robertson-Tessi M. et al., Acidity Promotes Tumour Progression by Altering Macrophage Phenotype in Prostate Cancer, British Journal of Cancer. (2019) 121, no. 7, 556–566, 10.1038/s41416-019-0542-2, 2-s2.0-85071029584. 31417189 PMC6889319

[50] Sauvant C. , Nowak M. , Wirth C. et al., Acidosis Induces Multi‐Drug Resistance in Rat Prostate Cancer Cells (AT1) in Vitro and in Vivo by Increasing the Activity of the P‐Glycoprotein via Activation of p38, International Journal of Cancer. (2008) 123, no. 11, 2532–2542, 10.1002/ijc.23818, 2-s2.0-57349151455. 18729196

[51] Lee H. S. , Park H. J. , Lyons J. C. , Griffin R. J. , Auger E. A. , and Song C. W. , Radiation-Induced Apoptosis in Different pH Environments in Vitro, International Journal of Radiation Oncology, Biology, Physics. (1997) 38, no. 5, 1079–1087, 10.1016/s0360-3016(97)00073-4, 2-s2.0-0030742976. 9276375

[52] Li W. , Zhou Y. , Shang C. , Sang H. , and Zhu H. , Effects of Environmental pH on the Growth of Gastric Cancer Cells, Gastroenterology Research and Practice. (2020) 2020, no. 1, 3245359–10, 10.1155/2020/3245359. 32211041 PMC7085403

